# Long-Term Quality of Life Outcomes After Laparoscopic Sleeve Gastrectomy and Roux-en-Y Gastric Bypass—a Comparative Study

**DOI:** 10.1007/s11695-020-05049-3

**Published:** 2020-10-16

**Authors:** Cristina Fiorani, Sophie R. Coles, Myutan Kulendran, Emma Rose McGlone, Marcus Reddy, Omar A. Khan

**Affiliations:** St George’s University Hospital, Blackshaw Road Tooting,, London, SW17 0QT UK

**Keywords:** Bariatric surgery, BAROS questionnaire, Sleeve gastrectomy, Gastric bypass, Comorbidity, Weight loss

## Abstract

Roux-en-Y gastric bypass (RYGB) and sleeve gastrectomy (SG) have been shown to improve metabolic comorbidities as well as quality of life (QoL) in the obese population. The vast majority of previous studies have investigated the metabolic effects of bariatric surgery and there is a dearth of studies examining long-term QoL outcomes post bariatric surgery. The outcomes of 43 patients who underwent bariatric surgery were prospectively assessed, using BAROS questionnaires to quantify QoL and metabolic status pre-operatively, at 1 year and at 8 years. Total weight loss and comorbidity resolution were similar between RYGB and SG. The RYGB cohort experienced greater QoL improvement from baseline and had higher BAROS scores at 8 years. RYGB may provide more substantial and durable long-term benefits as compared to SG.

## Introduction

Roux-en-Y gastric bypass (RYGB) and sleeve gastrectomy (SG) are well-established treatments for morbid obesity and have demonstrated particular efficacy in improving metabolic conditions such as type 2 diabetes mellitus (T2DM) and hypertension [1]. However in addition to their metabolic effects, there is evidence that these operations improve quality of life (QoL) via both weight loss–dependent and weight loss–independent effects [2]. To date, most studies comparing these procedures have focussed on metabolic effects as opposed to comparative QoL outcomes [3]. In addition, there are very limited long-term data on QoL outcomes post bariatric surgery.

The aim of this prospective study was to compare pre-operative, short-term and long-term QoL, weight loss and metabolic status in a consecutive cohort of patients undergoing RYGB and SG.

## Methods

Between May and October 2010, a total of 136 consecutive adult patients underwent RYGB (*n* = 92) or SG (*n* = 44) at St George’s Hospital, London (a tertiary referral centre for bariatric surgery) by three specialist bariatric consultant surgeons. Allocation to SG or RYGB was made by a multidisciplinary team based on patient and surgeon preferences. Surgery was performed in a standardised fashion—RYGB involved a formation of a small proximal gastric pouch with a bilio-pancreatic limb length of 60 cm and an alimentary limb of 100 cm. The SG was constructed in an antral-sparing fashion over a 34Fr Bougie.

### Data Collection

**Fig. 1:**
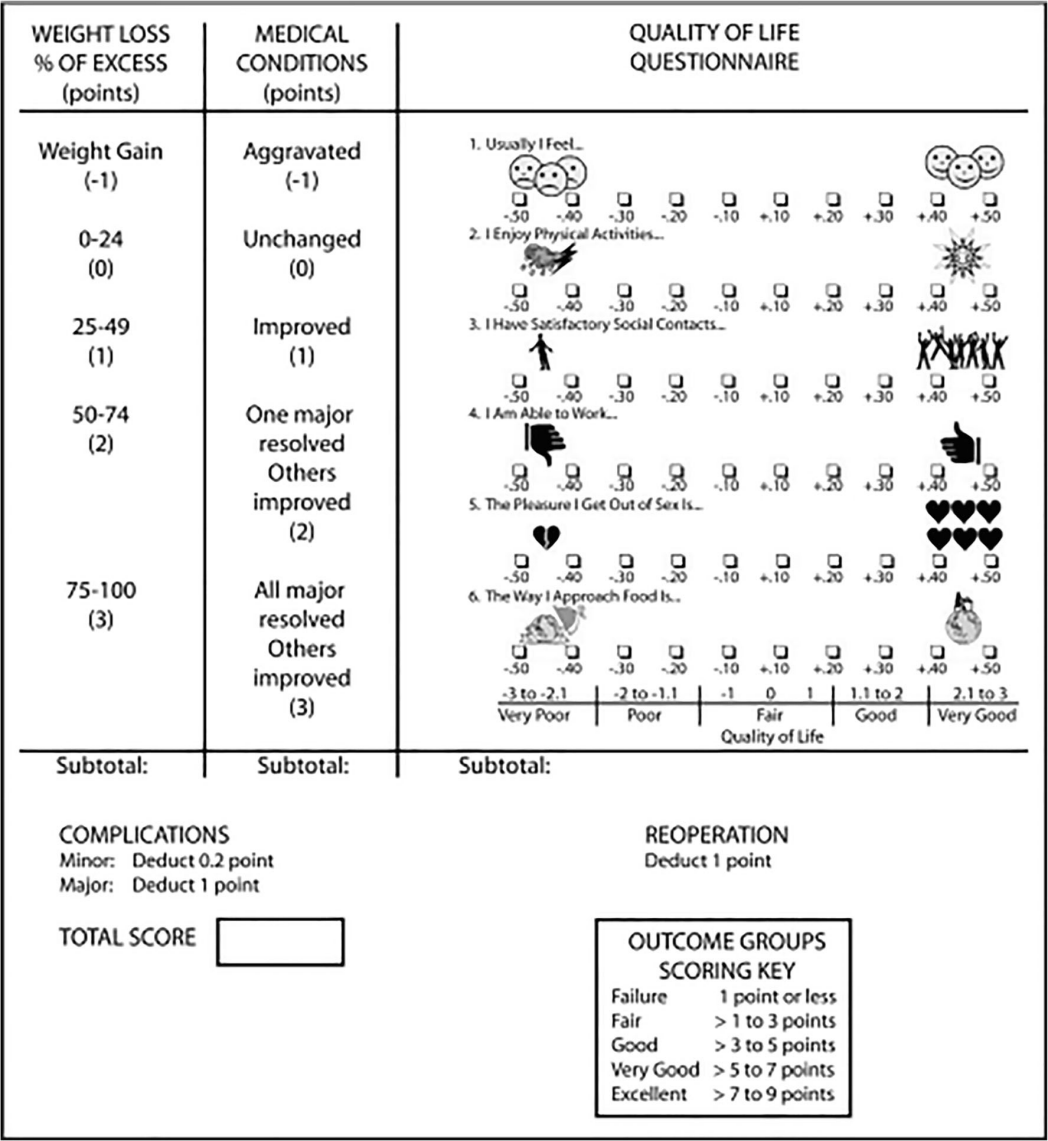
Summary of BAROS scoring system

The Bariatric Analysis and Reporting Outcome system (BAROS) was utilised as a validated tool to assess QoL and metabolic outcomes [3] (Fig. 1). For these purposes of this scoring system, resolution of T2DM was defined using the American Diabetes Association criteria (fasting blood glucose < 7.0 mmol/L and HbA1c < 6.5%). Improvement of any co-morbidity was defined as a reduction in the number of medications required.

Patients underwent pre-operative and 1-year post-operative assessments with face-to-face clinic appointments. At 8 years after surgery, the patients were contacted by post, telephone and email, and following contact with the patient, a telephone consultation was arranged. In order to increase the number in the cohort, non-responders were recontacted after 1 month by post, telephone and email (with the patients’ registered general practitioners contacted to check the accuracy of the patients’ contact details).

### Analysis

Of the initial cohort, 43 patients (RYGB = 32, SG = 11) completed the BAROS questionnaire at all three time points; and this group forms the basis of our study.

Data were expressed as mean ± standard error of the mean (SEM) with differences assessed using Student *t* tests or Mann-Whitney as indicated. Categorical data are presented as frequencies and percentages, with differences assessed using Fisher’s exact test or chi-square test as appropriate.

## Results

### Demographic Data

There were no significant differences in the mean pre-operative body mass index (BMI) or comorbidities between the two groups (Table 1).

**Table 1:** Demographic data, BMI, comorbidities and pre-operative QoL

		RYGB *n* = 32	SG *n* = 11	*p*
Age (years)		47 (8.3)	42.5 (3.62)	0.21
Sex (F^a^/M^b^)		27/5	10/1	1
Pre-op BMI^c^		45.6 (0.81)	47 (1.84)	0.43
Diabetes		11	4	1
Hypertension		15	2	0.15
OSA^d^		7	2	1
Dyslipidemia		9	2	0.70
GERD^e^		14	6	0.73
Baseline QoL scores based on BAROS [3]	Self-esteem	− 0.30 (0.03)	− 0.05 (0.06)	***< 0.001***
	Physical activity	− 0.25 (0.04)	0.02 (0.09)	***< 0.001***
	Social activity	− 0.23 (0.04)	− 0.03 (0.10)	***0.05***
	Work conditions	− 0.18 (0.05)	0.12 (0.11)	***0.01***
	Sexual activity	− 0.25 (0.03)	0.03 (0.08)	***< 0.001***
	Relationship to food	− 0.14 (0.04)	0.08 (0.04)	***0.01***
	**Total BAROS**	**− 1.35 (0.14)**	**0.17 (0.32)**	***< 0.001***

Values are expressed as mean (s.e.m.)

^a^*F* female, ^b^*M* male, ^c^*BMI* body mass index, ^d^*OSA* obstructive sleep apnea, ^e^*GERD* gastro-oesophageal reflux disease

### Quality of Life

Patients undergoing RYGB had poorer pre-operative QoL in all domains as compared to SG (Table 1).

### Peri-operative Complications

There were no major complications (Clavien-Dindo grade 3 or higher), and no patients underwent revisional surgery during the follow-up period.

### Weight Loss

There was no significant difference in weight loss parameters between the two groups (Table 2).

**Table 2:** Weight loss and changes in comorbidities and in QoL outcomes post RYGB and SG

Weight		RYGB	SG	*p*
1 year	BMI^a^	37.7 (0.79)	39.7 (1.87)	0.27
	Change in BMI^a^	7.9 (0.46)	7.3 (0.75)	0.54
	Total weight loss (%)	17.2 (0.97)	15.7 (1.57)	0.42
	EBMIL^b^ (%)	39.0 (2.35)	35.1 (3.86)	0.40
8 years	BMI^a^	32 (0.99)	33.6 (1.75)	0.43
	Change in BMI^a^	13.6 (0.94)	13.4 (2.23)	0.93
	Total weight loss (%)	29.2 (1.87)	27.7 (4.16)	0.69
	EBMIL^b^ (%)	66.8 (4.65)	59.4 (8.41)	0.43
Quality of life scores		RYGB	SG	*p*
1 year	Self-esteem	0.28 (0.03)	0.24 (0.07)	0.49
	Physical activity	0.19 (0.04)	0.20 (0.07)	0.88
	Social activity	0.25 (0.08)	0.22 (0.07)	0.65
	Work conditions	0.18 (0.05)	0.25 (0.07)	0.44
	Sexual activity	0.10 (0.06)	0.13 (0.10)	0.80
	Relationship to food	0.28 (0.04)	0.27 (0.08)	0.98
	Total	1.27 (0.18)	1.31 (0.40)	0.92
8 year	Self-esteem	0.22 (0.04)	0.11 (0.07)	0.19
	Physical activity	0.10 (0.05)	0.02 (0.07)	0.37
	Social activity	0.25 (0.05)	0.25 (0.07)	0.96
	Work conditions	0.29 (0.06)	0.34 (0.09)	0.68
	Sexual activity	0.09 (0.06)	−0.03 (0.12)	0.35
	Relationship to food	0.25 (0.05)	0.24 (0.07)	0.88
	Total	1.24 (0.21)	0.93 (0.30)	0.45
Comorbidity		RYGB	SG	*p*
Resolved or improved *n* (%)	T2DM^c^	6 (55)	2 (50)	n/a ^*^
	Hypertension	6 (40)	0	n/a ^*^
	OSA^d^	5 (71)	1 (50)	n/a ^*^
	Dyslipidemia	4 (44)	1 (50)	n/a ^*^
	GERD^e^	5 (36)	0	n/a ^*^

Values are expressed as mean (s.e.m.)

^*a*^*BMI* body mass index, ^b^*EBMIL* excess body mass index loss, ^c^*T2DM* type 2 diabetes mellitus, ^d^*OSA* obstructive sleep apnea syndrome, ^e^*GERD* gastro-oesophageal reflux disease

^*^n/a non-applicable—due to the small numbers *p* value was not calculated

### Co-morbidities

Changes in pre-operative comorbidities 8 years after surgery are displayed in Table 2.

Of 11 patients with pre-operative T2DM in the RYGB cohort, six of whom required insulin, two experienced resolution of their T2DM and four no longer required insulin; of four patients with pre-operative T2DM in the SG cohort, none of whom required insulin, two resolved.

### Quality of Life Changes

Detailed analysis of QoL scores revealed that all components of the QoL score were significantly improved at 1- and 8-year follow-up in the RYGB cohort as compared to pre-operative scores.

In the SG cohort, there were significant improvements only in self-esteem (*p* = 0.03) and social activity (*p* = 0.01) scores at 1-year follow-up as compared to pre-operative scores.

At 8 years, only social activity scores remaining improved (*p* = 0.03).

Despite patients undergoing RYGB having worse QoL scores at baseline, QoL scores at 1 year between the two groups were comparable. RYGB group achieved a greater improvement in QoL outcomes at 8 years (Table 2).

### Overall BAROS Scores

Mean BAROS scores were similar between the two groups at 1 year (3.49 ± 0.24 vs 3.49 ± 0.51; *p* = 1.00) but were significantly higher in the RYGB group at 8 years (2.64 ± 0.32 vs. 1.47 ± 0.36; *p* = 0.02).

## Discussion

Given obesity is a chronic, multifaceted disease, there is a need to evaluate long-term QoL outcomes following bariatric surgery. This is one of the first prospective studies reporting longer-term QoL outcomes of patients undergoing RYGB and SG. Our results demonstrate good QoL, weight loss and metabolic outcomes at 1 and 8 years, with none of the patients requiring emergency or revision surgery (as compared to reported re-operative rates of up to 7% [4]). It should be noted, however, that the final BAROS scores in our cohort appear to be less favourable than those described in the few previously published longer-term studies evaluating BAROS outcomes after RYGB and SG [5–9]. This finding may be partly due to the shorter follow-up in these studies and that the cohort in these studies were less obese and hence would be expected to have higher BAROS scores at follow-up [8].

Long-term weight loss was similar between RYGB and SG—a finding consistent with previous randomised studies [1, 2]. Comorbidity resolution rates also appeared broadly comparable—although the very small numbers preclude statistical analysis and hence definitive conclusions on this subject. With regard to quality of life, scores improved in all domains after RYGB at 8-year follow-up, compared to improvement in only social activity scores in the SG cohort only. Although QoL and overall BAROS scores at 1 year were similar between the two cohorts, the RYGB cohort had higher scores at 8 years. These results suggest that RYGB has both a more profound and a more durable effect on quality of life than SG.

We acknowledge that the high incidence of loss to follow-up (though comparable to studies with similar duration [10]) and the relatively small size of our cohort does limit the strength of the conclusions we can draw. In particular, we were unable to analyse any potential interactions between reduction in body weight, resolution of co-morbidities and improvement in self-reported QoL. In addition, we accept that this was not a randomised control study; however, the fact that RYGB cohort group had more adverse initial clinical features but still had better long-term outcomes in fact adds validity to our conclusions on the relative merits of the two procedures.

## Conclusions

RYGB appears to provide more substantial and durable long-term benefits for morbidly obese patients as compared to SG particularly in terms of QoL improvements. These results require validation in a larger study.
